# Inhibition of glutamine metabolism: acting on tumoral cells or on tumor microenvironment?

**DOI:** 10.18632/oncotarget.28443

**Published:** 2023-08-10

**Authors:** Raul Peña

**Keywords:** glutamine, tumor microenvironment, fibroblasts, CAF, snail1

Cancer cell growth and survival relies in metabolites and metabolic routes different than those used by healthy cells. Glucose and glutamine (Gln) uptake and consumption is increased by many cancer types in order to support their high growth rate [1]. Besides being metabolized to tricarboxylic acid (TCA) cycle precursors, Gln is necessary also for the generation of nitrogen-containing metabolites, such as nucleotides, glucosamine-6-phosphate or nonessential amino acids. Indeed, nitrogen supply has been widely described as limiting for cell cycle progression.

As mitochondrial glutaminase (GLS) directs Gln into the TCA cycle, its inhibition has been suggested as a potential strategy for targeting and blocking Gln metabolism in cancer cells. In fact, GLS inhibitors block cancer cell growth *in vivo* and *in vitro*. Based on this premise, several clinical studies have been conducted to test if Gln dysregulation increase cancer patients’ survival [2]. So far, these treatments have not been able to induce a great overall benefit for patients due to the ability of tumor cells to alter their metabolism. Different authors have described an increase in the oxidative stress after alterations in Gln metabolism *in vivo* [3], suggesting the possibility to combine glutamine dysregulation strategies with some other therapies increasing reactive oxidative species to promote cancer cell death.

Interestingly, treatments with GLS inhibitors have been used to improve the outcome of several diseases caused by fibroblasts in preclinical studies. For example, during liver fibrosis GLS1 levels and Gln metabolism are increased during the course of the disease. Treatments to block GLS1 activity alleviate fibroblasts activation, secretion of inflammatory cytokines and, therefore, fibrosis *in vivo* [4]. Similar results were described in pulmonary fibrosis [5]. The authors proposed a molecular mechanism by which a classical activator of fibroblasts, TGFβ, promotes profibrotic gene expression in a GLS1 dependent manner. Accordingly, in a murine model of bleomycin-induced lung fibrosis *in vivo*, a blockade in Gln metabolism reversed lung inflammation, fibrosis and restored oxygen saturation in blood.

Recently, we described a new action of Gln on cancer-associated fibroblasts (CAFs) in breast cancer [6]. CAFs are the main cellular component of tumor microenvironment (TME), defined as the complex environment in which tumor cells grow, and composed by cells, soluble molecules and extracellular matrix. In our study, we determined that mesenchymal-like epithelial breast tumor cells and CAFs present a higher dependence on Gln than tumor epithelial breast cancer cells. This originates that CAFs migrate from low-to high Gln regions. Thus, when CAFs were challenged *in vitro* with a Gln gradient, they migrated and invaded towards the Gln-high compartment. This effect required their previous Snail1-dependent activation by TGFβ or by other factors derived from the cancer cells. Moreover, since CAFs can cooperate with tumor epithelial cells, migration of these cells was also stimulated *in vitro* when they were co-cultured with activated fibroblasts in a Gln gradient. Similar effect was obtained *in vivo* when we implanted a Gln-soaked plug in one side of an ectopically generated breast tumor: epithelial cells migrated preferentially toward this plug instead to PBS soaked plugs.

Molecularly, Gln-directed CAF-migration was associated to an Akt2 redistribution to the leading edge of the cell. This polarized subcellular distribution depends on TRAF6 and on p62/SQSTM1 as fibroblasts with impaired TRAF6/p62 protein expression lose their ability to redistribute active Akt2 and therefore, to properly migrate.

**Figure 1:**
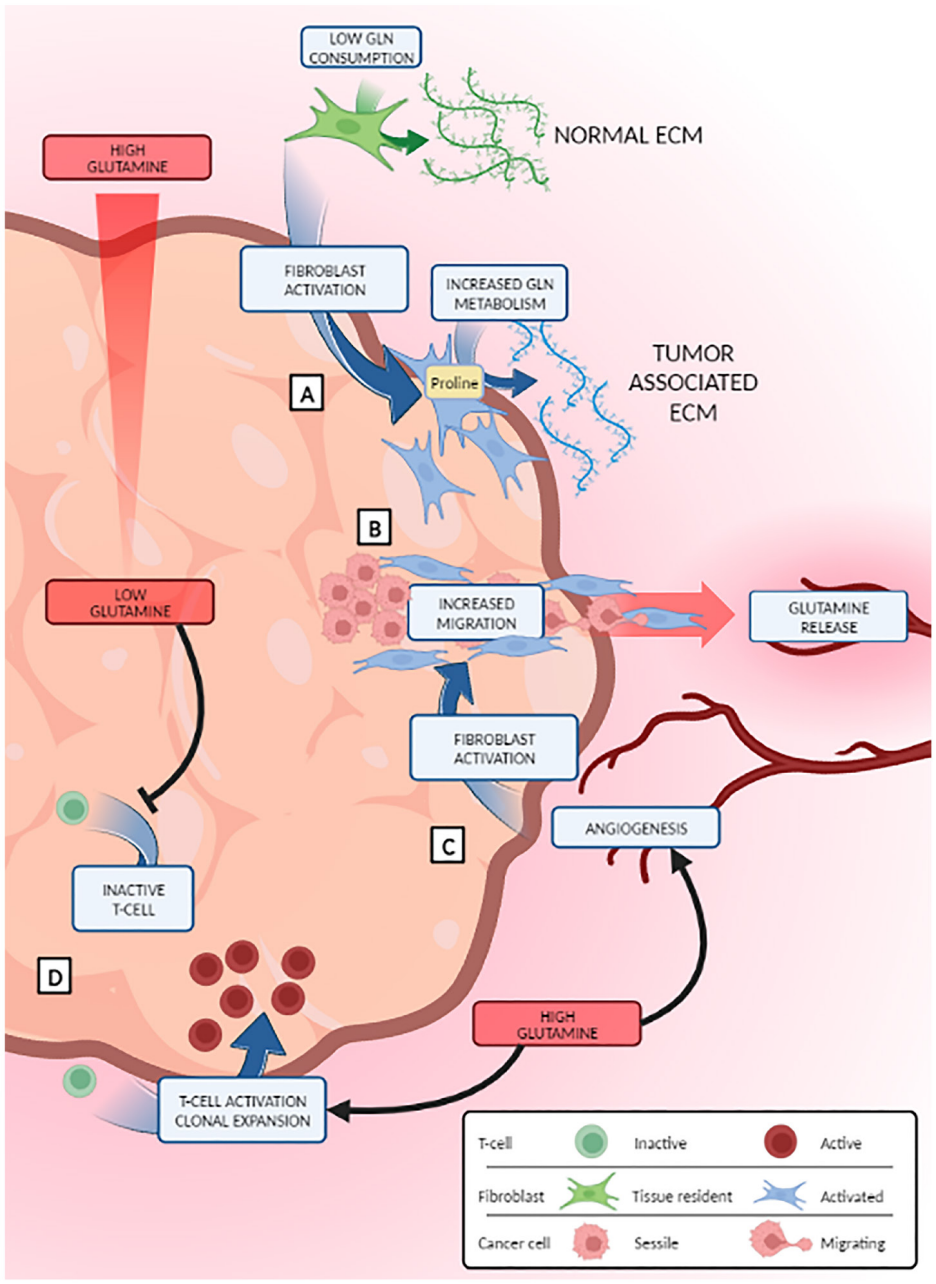
Glutamine metabolism effect in tumor microenvironment cells. Tumor growth creates a glutamine gradient ranging from lower levels of the metabolite in the core of the tumor to more elevated concentrations at the periphery, specially, close to neo-vessels (represented with the red arrow and background). Normal fibroblasts, upon activation, convert glutamine into proline to produce tumor extracellular matrix (**A**) and migrate from the inner part of the tumor towards the Gln-rich margin helping tumor cells to metastasize (**B**). Moreover, neo-vessels release glutamine attracting activated fibroblasts towards blood vessel and therefore increasing metastasis (**C**). Glutamine deprivation also impairs immune cells action, as T-cells fail to be activated in the low glutamine areas of the central parts of the tumor (**D**).

In summary, we proposed a novel and interesting mechanism by which Gln, usually concentrated at the tumor periphery, acts as a chemoattractant for CAFs, enhancing extracellular matrix degradation and facilitating epithelial cancer cell migration and metastasis *in vivo* (Figure 1).

Interestingly, we have also described a model in which endothelial cells activation by cancer cells, besides being a source of tumor associated neo vessels also enhances fibroblast activation in breast tumors [7]. As the main delivery of Gln to the tumors is the bloodstream, it is easy to speculate that the migration of endothelium-activated fibroblast towards the glutamine gradient created around newly formed capillaries facilitates tumor cancer cells metastasis.

A recent report describes an alternative strategy by which cancer cells are boosted by CAFs glutaminolysis in breast cancer. In this model, *de novo* synthesis of proline (Pro) from Gln is used by CAFs to produce and deposit more collagen in the extracellular matrix [8]. By reducing the expression levels of Pyrroline-5-carboxylate reductase (Pycr1) in CAFs, a key enzyme for Pro synthesis, the tumor reduced collagen content and exhibited a decrease in growth and metastasis *in vivo*, reinforcing the idea that Gln metabolism is key for the pro-tumoral role of CAFs (Figure 1).

Other reports have suggested altered roles for other TME cells in response to GLS inhibition. Actually, an increase in Gln consumption is associated not only to cell transformation but to normal cellular processes where energy or nitrogen compounds are highly demanded, such as T-cell activation [9] or endothelial cell angiogenesis [10]. Different cell types in TME react in different ways to Gln deprivation acquiring new pro- or anti-tumoral roles (Figure 1). CD4+ and CD8+ T-cells activation is impaired after GLS inhibition in several models of lung cancer, suggesting that inhibition of Gln metabolism promotes a local immunodepression and a pro-tumoral effect in advanced cancer stages [11, 12]. Nevertheless, other authors found that endothelial-specific lack of GLS induced vascular normalization, decreased hypoxia, and inhibited tumor growth and metastasis in a breast cancer model [13]. Their findings, associated with an increased delivery and sensitivity of cancer cells to chemotherapeutic agents, suggest an overall antitumorigenic effect upon Gln metabolism disruption.

These recent publications reveal the complexity of TME and cancer cells interplay. This process is key in cancer progression and should be properly understood to better fight against the disease. Recent research has highlighted the effects of Gln deprivation in tumors that does not just affect cancer cells but also to the entire TME. These new findings evidence the necessity of more research in Gln metabolism to define the best therapeutic strategies for cancer patients.
